# Co-relation with novel phosphorylation sites of IκBα and necroptosis in breast cancer cells

**DOI:** 10.1186/s12885-021-08304-7

**Published:** 2021-05-24

**Authors:** Sung Hoon Choi, Hee-Sub Yoon, Shin-Ae Yoo, Sung Ho Yun, Joo-Hee Park, Eun Hee Han, Sung-Gil Chi, Young-Ho Chung

**Affiliations:** 1 Research Center for Bioconvergence Analysis, Korea Basic Science Institute (KBSI), 162 Yeongudanji-ro, Ochang-eup, Cheongwon-gu, 28119 Cheongju-si, Republic of Korea; 2 Yonsei Liver Center, Yonsei University College of Medicine, Seoul, 03722 South Korea; 3 Department of Life Sciences, Korea University, Seoul, 02841 Republic of Korea; 4 GRAST, Chungnam National University, Daejeon, 34134 Republic of Korea

**Keywords:** Breast cancer, IκBα, New phospho-site, Necroptosis

## Abstract

**Background:**

Phosphorylation of NF-kappaB inhibitor alpha (IκBα) is key to regulation of NF-κB transcription factor activity in the cell. Several sites of IκBα phosphorylation by members of the IκB kinase family have been identified, but phosphorylation of the protein by other kinases remains poorly understood. We investigated a new phosphorylation site on IκBα and identified its biological function in breast cancer cells.

**Methods:**

Previously, we observed that aurora kinase (AURK) binds IκBα in the cell. To identify the domains of IκBα essential for phosphorylation by AURK, we performed kinase assays with a series of IκBα truncation mutants. AURK significantly promoted activation of IκBα at serine 32 but not serine 36; by contrast, IκB kinase (IKK) family proteins activated both of these residues. We also confirmed phosphorylation of IκBα by matrix-assisted laser-desorption/ionization time-of-flight mass spectrometry (MALDI-TOF/TOF MS) and nano-liquid chromatography hybrid quadrupole orbitrap mass spectrometer (nanoLC-MS/MS; Q-Exactive).

**Results:**

We identified two novel sites of serine phosphorylation, S63 and S262. Alanine substitution of S63 and S262 (S63A and S262A) of IκBα inhibited proliferation and suppressed p65 transcription activity. In addition, S63A and/or S262A of IκBα regulated apoptotic and necroptotic effects in breast cancer cells.

**Conclusions:**

Phosphorylation of IκBα by AURK at novel sites is related to the apoptosis and necroptosis pathways in breast cancer cells.

**Supplementary Information:**

The online version contains supplementary material available at 10.1186/s12885-021-08304-7.

## Background

Breast cancer is the most frequent malignant tumor in women and the leading cause of cancer death, since 30% of breast cancers develop distant metastases after the initial treatment of the apparently localized tumors [1]. Nowadays, the mechanisms underlying the genesis and progression of breast cancer are better understood [2, 3], but despite an improvement of the survival rates for breast cancer, we still have to go a long way to know the cure for all patients [2, 4].

Protein-protein interactions (PPI) mean that two or more proteins bind each other, to perform their biological functions [5]. Most of the important molecular processes in the cell, such as DNA replication, are performed by large molecular network of protein-protein interactions [6]. Those are built of a large number of protein elements organized by their PPI [5, 6]. Also, Interactions of proteins are important for many biological functions [7]. For example, external signals are transmitted to the interior of the cell by PPI [6, 7]. This process, called signal transduction, plays the essential role in many biological processes and diseases, such as cancer. Protein-protein interactions are the primary mechanism for virtually every process in a living cell [7]. We need to acquire more knowledge of various protein-protein interactions in order to understand biological phenomena, including diseases, and to provide the basis for new therapeutic approaches.

IκBα (nuclear factor of kappa light polypeptide gene enhancer in B-cells inhibitor, alpha) is a member of a family of cellular proteins to inhibit the NF-κB transcription factor [8]. Furthermore, IκBα blocks the ability of NF-κB transcription factors binding to DNA, which is required for proper function of NF-κB [9]. However, NF-κB activation signal induce the activation of a putative protein-tyrosine phosphatase(s), leading to I kappa B-alpha serine 32/36 phosphorylation and degradation and NF-κB nuclear translocation [10]. Some Hodgkin lymphoma cells have the mutated gene part of encoding the protein IκBα [11]. These mutations inactivate the IκBα protein [11]. Thus, NF-κB to become active on the lymphoma tumor cells chronically, this activity contributes to the malignant state of these tumor cells [8, 9, 12]. On the other hand, Inhibition of NF-kappaB/Rel induces apoptosis of variable normal or cancer cells [13].

AURK protein is one member of the Aurora subfamily of serine/threonine protein kinases that are essential for cell proliferation [14]. Aurora kinase subfamily comprise three varieties; Aurora-A, Aurora-B and Aurora-C. AURKA and AURKB are involved in mitosis (cell division producing identical daughter cells), and AURKC is involved in meiosis (sexual reproduction) [14, 15]. These proteins is over-expressed in various cancer cell lines such as breast cancer [16, 17], suggesting an involvement in oncogenic signal transduction [17].

In previous work, we identified a novel PPI between AURKC and IκBα [17]. In this study, using MALDI-TOF MS and nanoLC-quadrupole orbitrap MS/MS, we detected new sites of AURKC-mediated phosphorylation in IκBα. To elucidate the intracellular functions of the new phosphorylated residues, we performed a series of experiments to measure proliferation and apoptosis in breast cancer cell lines.

## Methods

### Proteins

Active proteins (IKKα, IKKβ and Aurora kinase C; Signalchem, Vanier Place Richmond, BC, Canada), kinase buffer I and ATP stock solution were purchased from signalchem. Halo-Tag plasmid vector (pFN18A), Restriction enzyme blend (SgfI & PmeI), KRX expression cell, L-rhamnose monohydrate and sequencing-grade modified trypsin were purchased from Promega (Madison, WI). Quikchange lightning site-directed mutagenesis kit was purchased from Agilent technologies. D- glucose and phosphoric acid (PA) were purchased from Sigma-Aldrich. 2,5-Dihydroxybenzoic acid (2,5-DHB) was purchased from Bruker Daltonics. Acetonitrile (ACN, ultrapure ACS reagent grade and methyl alcohol (high purity HPLC reagent) were purchased from USB corporation and samchun pure chemical. All other chemicals used were of ACS or HPLC grade. Primary antibody (anti-IκBα monoclonal antibody) and anti-mouse secondary antibody were purchased from Santa Cruz Biotechnology and Cell signaling Technology. Primary antibody (anti-phospho-IκBα serine 32; Cell Signaling Technology, CA, USA, serine 63 and serine 262 monoclonal antibody; Custom made antibody) and anti-rabbit secondary antibody were purchased from abcam and Cell signaling Technology. Primer and DNA sequencing data were purchased cosmo genetech (Seoul, Korea).

### Cloning and mutagenesis of IκBα

IκBα constructs were cloned into the Halo-Tag vector pFN18A (Promega) for *E.coli* overexpression between the restriction sites SgfI and PmeI. Mutagenesis reactions of the IκBα single and double point mutant were performed with the Quikchange lightning site-directed mutagenesis kit (Agilent technologies). The following oligonucleotides were used as sense primers: S32A, 5′-CACGACGCCGGCCTG-3′; S36A, 5′-CTGGACGCCATGAAA-3′; S32/36A, 5′-CACGACGCCGGCCTGGACGCCATGAA-3′. The C-terminal deletion mutants were by insertion of new GAAGCAGCAGCTCACCGAGTGAGTTTAAACGAATTCGGGCT-3′;CGCACCTCCACTCCATCTGAGTTTAAACGAATTCGGGCT-3′; IκBα stop codon with following PCR antisense primers: IκBα 1–72, 5′-1-175,1–277,CTGACACTAGAAAACCTTTGAGTTTAAACGAATTCGGGCT-3′. The construction of the ankyrin repeat domain deleted IκBα 17 was used overlap PCR method with following PCR antisence and sense primers: IκBα 1–72/278–317, 5′-GAAGCAGCAGCTCACCGAGCAGATGCTGCCAGAGAGTG-3′, 5′-GAAGCAGCAGCTCACCGAGCAGATGCTGCCAGAGAGTG-3′. The mutated PCR fragments also were cloned into *E.coli* overexpression vector pFN18A (Promega).

### Overexpression of IκBα in KRX cell

Full-length human IκBα was cloned into a pFN18A vector (Promega). The plasmid is transformed into KRX cell (Promega) grown in LB media (add to ampicillin) supplemented with glucose and rhamnose to induce expression without isopropyl-β-D-thiogalactoside (IPTG). After growing the cells to OD 600 of 0.5–0.6 at 37 °C, the temperature was reduced to 20 °C for overnight expression.

### Purification of IκBα

For IκBα purification, cell pellets were resuspended in 5 mL of HaloTag purification buffer (50 mM HEPES (Sigma-aldrich), pH 7.5 and 150 mM NaCl) supplemented with 1x Protease Inhibitor (Thermo scientific), and sonicated on ice using a vibra cell sonicator (5 min total time on; 1 min on / 1 min off; amplitude = 40–60, pulse = 4). Lysates were centrifuged at 10,000×g for 30 min at 4 °C and the supernatants were directly applied onto pre-equilibrated HaloLink resin (Promega) following manufacturer recommendations. Binding to the resin was conducted at room temperature for 1 h with constant end-over-end rotation, followed by three washes, each for 5 min with 10 mL purification buffer. Target proteins were released from the resin by proteolytic cleavage using a ratio of HaloTEV to settled resin in HaloTag purification buffer for 1 h at room temperature. The supernatants containing the released protein of interest and TEV protease were carefully removed into new tube. For TEV protease removal, add of HisLink resin into the tube. Binding to the resin was conducted at room temperature for 20 min with constant end-over-end rotation. Spin at 1000×g for 5 min, and transfer supernatant to another tube. Protein quantification was conducted by the Bradford method [10]. Bovine serum albumin was used for the calibration of sample protein quantity.

### Cell culture

MCF-7 (KCLB60104, Korean Cell Line Bank) and MDA-MB 231 (KCLB88064, Korean Cell line Bank) cells were cultured at 37 °C with 5% CO2 in Dulbecco’s Modified Eagle Medium (DMEM; Gibco, Grand Island, NY) supplemented with 10% fetal bovine serum (FBS; Gibco, CA, USA), 4.5 g/L glucose, L-glutamine, and 1% penicillin/streptomycin.

### Staining or Western blot analysis

For in-gel trypsin digestion, the gels were stained by Coomassie brilliant Blue-R250 (CBB, 3 M co.). For deletion mutant of IκBα size visualization, the gels were stained by silver staining (GE Healthcare, uppsala, Sweden). For Western blotting, the protein separated by electrophoresis were transferred to 0.2 μm Polyvinylidene fluoride (PVDF; Bio-Rad Laboratories Inc.)membranes in Transfer buffer (25 mM Tris, 192 mM Glycine, 20% (v/v) methanol) at 48 mA for 12 h using Mini Trans-Blot Cell system (Bio-Rad Laboratories Inc.). in order to confirm the protein expression level as aging is processing, the transferred membrane was incubated in 5% (W/V) skim milk with 50 mM Tris-buffered saline with Tween 20 (TBST, Intron biotechnology, 24.7 mM Tris, pH 7.8, 2.7 mM KCl, 137 mM NaCl and 0.05% Tween-20) for blocking, and exposed to primary antibody (RIP3, MLKL, Caspase-3, Caspase-8, beta-actin: Cell Signaling Technology, CA, USA) overnight at 4 °C. After washing step (three times for 15 min with fresh TBST), the membrane was exposed to secondary antibody for 2 h at room temperature. After washing step, the proteins were visualized using ECL solution and detected by ChemiDoc XRS+ system (Bio-Rad Laboratories Inc.). The transcription activity of p65 was measured by enzyme-linked immunosorbent assay (ELISA; R&D Systems, Mineapolis, MN, USA), performed according to the manufacturer’s instructions. In the NF-κB/p65 assay, a double stranded DNA sequence containing the NF-κB response element was immobilized to the bottom of the wells of a 96-well plate. NF-κB contained in the nuclear extract bound to the NF-κB response element, and was then detected with anti–NF-κB p65 antibody. A secondary antibody conjugated to HRP was added to provide a colorimetric readout at 450 nm.

### In vitro kinase assay

Recombinant active AURK (100 ng) (Signalchem, BC, Canada) was incubated with 10 μM ATP (Signalchem, BC, Canada), 20 μl kinase buffer (25 mM HEPES, 25 mM β- glycerophosphate, 25 mM MgCl2, 2 mM dithiotreitol, and 0.1 mM NaVO3) and purified inactivated IκBα. Reactions were incubated at 37 °C for 30 min and terminated by addition of Laemmli SDS sample dilution buffer (Bio-rad, CA, USA). Proteins were separated by SDS-PAGE, and phosphorylation was detected specific antibody.

### Cell growth assay

Cell Growth Assay. Breast cancer cells growth rates were measured using the WST-1 (2-(2-methoxy-4-nitrophenyl)-3-(4-nitrophenyl)-5-(2,4-disulfophenyl)-2H-tetrazolium (WST-1, Sigma-Aldrich, CA, USA) method. First, the cells were seeded in a 96-well plate at 5 × 103 cells/well, incubated at 37 °C for 24 h, transferred to serum-free medium, and transfected with S63A and S262A as described above. Then, after cultivation under 21% O2 for 24 h, the cells were transferred to a culture medium containing 10% FBS. Finally, WST-1 was added to the wells, and the plates were incubated at 37 °C for 3 ~ 4 h for efficient cell dyeing, and analyzed for its absorbance at 460 nm using a spectrophotometer (Molecular Devices, USA). And, cell viability was measured by tryphan blue staining. It is based on the principle that live cells possess intact cell membranes that exclude certain dyes, such as trypan blue. When a cell suspension is simply mixed with the dye, add equal parts of 0.4% trypan blue dye to the cell suspension to obtain a 1 to 2 dilution, and incubate mixture for less than 3 min at room temperature. Place the hemacytometer with 1:1 tryphan blue staining cells on the stage of a light microscope (Olympus Optical Co., Tokyo, Japan) and calculated the staining cells.

### Cell death assay-FACs

FACs Cells were stained with FITC-labeled annexin V and propidium iodide, and tumor cell death was assessed by terminal deoxynucleotidyl transferase dUTP nick end labeling (TUNEL; Millipore, Billerica, MA, USA) assay and flow cytometry (BD- Flow JO).

### In gel trypsin digestion

The in-gel digestion of phosphorylated IκBα was excised from the polyacrylamide gel, washed with water and 30% methanol, and destained with 50% acetonitrile/10 mM ammonium bicarbonate (ABC) at room temperature until the CBB color was removed. The gel pieces were dehydrated with 100% (v/v) acetonitrile. After drying the organic solvent using Speed vacuum concentrator. Disulfide bonds were reduced with DTT (10 mM, 56 °C, 1 h), and the free sulfhydryl groups were alkylated with iodoacetamide (55 mM, room temperature, 40 min in the dark). The gels were vortexed and completely dried in Speed vacuum concentrator. Gel pieces were washed with water, and dehydrated with 100% acetonitrile. After drying with a Speed vacuum concentrator, the gel was rehydrated using 100 ng/μl trypsin (50 mM ABC, pH 8.3) and the digestion was carried out at 37 °C for the maximum 20 h for full digestion. The tryptic peptides were collected in extraction steps using 50% (v/v) acetonitrile containing 0.1% (v/v) formic acid. The tryptic peptide extracts were pooled and dried in Speed vacuum concentrator. The peptides were redissolved in 1% phosphoric acid for mass spectrometric analysis.

### Mass spectrometry

Phosphorylation of IκBα were analyzed by both matrix-assisted laser desorption/ionization time-of-flight (MALDI-TOF) and nanospray-liquid chromatography hybrid quadrupole-Orbitrap mass spectrometer (nanoLC-Q Exactive Plus). For MALDI MS, 0.5 μl of sample and 0.5 μl of matrix solution were mixed followed by sample mixture was spotted onto a stainless-steel MALDI plate. The matrix solution was prepared by supernatant from a saturated solution of 2,5-dihydroxybenzoic acid (2,5-DHB) with 50% acetonitrile/1% phosphoric acid [12]. The spots were analyzed with the ultrafleXtreme™ MALDI TOF-TOF mass spectrometer (Bruker Daltonics). MS spectra were collected in reflectron mode over the set mass range of *m/z* = 700–3500. Instruments were calibrated using Bruker peptide standard II. For LC-MS/MS, tryptic peptides were loaded on a trapping column with 75 μm inner diameter, packed with 5 μm C18 particles (Acclaim PepMap100, Thermo Scientific) and analyzed using a 15 cm analytical column packed with 2 μm C18 particles (Acclaim PepMap RSLC, Thermo Scientific). Reversed phase chromatography was performed using an Ultimate 3000 RSLC nano system (Thermo Scientific) with a binary solvent consisting of 0.1% formic acid (buffer A) and 80% ACN in 0.1% formic acid (buffer B). The peptides were separated by a linear gradient of buffer B from 5% up to 95% for 180 min with a flow rate of 300 nl/min. The LC was coupled to a Q Exactive hybrid quadrupole-Orbitrap mass spectrometer (Q Exactive Plus, Thermo Scientific). The Q Exactive Plus was operated in data-dependent mode with MS scans acquired at a resolution of 70,000, an ion target value of 1e6, and maximum ion injection time for the MS scan was set to 250 ms. Up to 15 most abundant isotope patterns with charge ≥2 from the survey scan were selected for MS/MS. An isolation window of 2.0 m/z and higher energy collisional dissociation (HCD) with normalized collision energies of 27% was applied. The maximum ion injection time for the MS/MS scans was set to 100 ms and the ion target value to 1e5. Repeated sequencing of peptides was kept to a minimum by dynamic exclusion of 40 s [13, 14].

### Data analysis

Measured MS data were processed using FlexAnalysis (Bruker Daltonice). MS/MS data acquired with the Q Exactive was searched the human IκBα FASTA using Mascot. Search parameters included two missed cleavages from trypsin proteolysis and fixed modification of cysteine residues. Variable modifications included oxidation (M), and phosphorylation (S and T). The ion-score cutoff was less than zero or 10^− 5^ at significance threshold *p* < 0.05.

### Statistical analysis

Results were expressed as means ± standard error of the mean (SEM) or frequency (%). An independent t-test was performed to compare the difference of the means between control and experimental groups. All statistical analysis was done using SPSS version 12.0 (SPSS, Inc., Chicago, IL). A *p* value of less than 0.05 was considered statistically significant.

## Results

### Analysis of phosphopeptides in IκBα by AurkC using MALDI-TOF MS

In a previous study [17], we identified an interaction between IκBα and AURKC using the CUPID system. The observation was confirmed by conventional experiments, including co-immunoprecipitation and the mammalian two-hybrid system. The PPI between IκBα and AURKC, which are both serine/threonine kinases, leads to IκBα phosphorylation. Interestingly, although IκBα is known to be phosphorylated at S32 and S36, AURKC phosphorylates IκBα only at S32; however, the phospho-band was thick and shifted, implying that it represented novel phosphorylation sites [17]. To identify the carcinogenic mechanism of IκBα–AURKC binding, we screened IκBα phosphorylation sites using MALDI-TOF MS and nanoLC-quadrupole orbitrap MS/MS, and detected a new IκBα phosphorylation site with AURKC (Supporting Fig. 1). Phospho-IκBα was enriched to analyze phosphopeptides by MS. Instead of dissolving 2,5-dihydroxybenzoic acid matrix in a phosphoric acid (PA) solution, we used an alpha-cyano-4-hydroxycinnamic acid matrix in trifluoroacetic acid, a method commonly employed in peptide analysis. To identify the specific site of phosphorylation of IκBα by AURKC, we used IκBα phosphorylated by IKKβ and unphosphorylated IκBα as negative controls. We detected 11 of 22 tryptic peptides in phosphorylated IκBα using MALDI-TOF MS and also identified three phosphorylated peptides. The tryptic peptides, containing missed cleavages and modified peptides, including oxidized (+ 16 Da) and carbamidomethylated (+ 57 Da) IκBα, are confirmed with their theoretical molecular weights. The mass of a phospho-peptide increases by ~ 80 Da due to the binding of HPO_3_^−^ to serine or threonine. An increase of 80 Da in peptide T6 (amino acids 30–38), which contains S32, was observed at *m/z* 1069.40 (P1). We had anticipated that the original T6 peptide (989.44 Da) would be phosphorylated at S32. However, the original T6 peptide peak could not be detected because it underwent various modifications and missed cleavages (Supplementary Fig. 2 and Table 1).

**Fig. 1:**
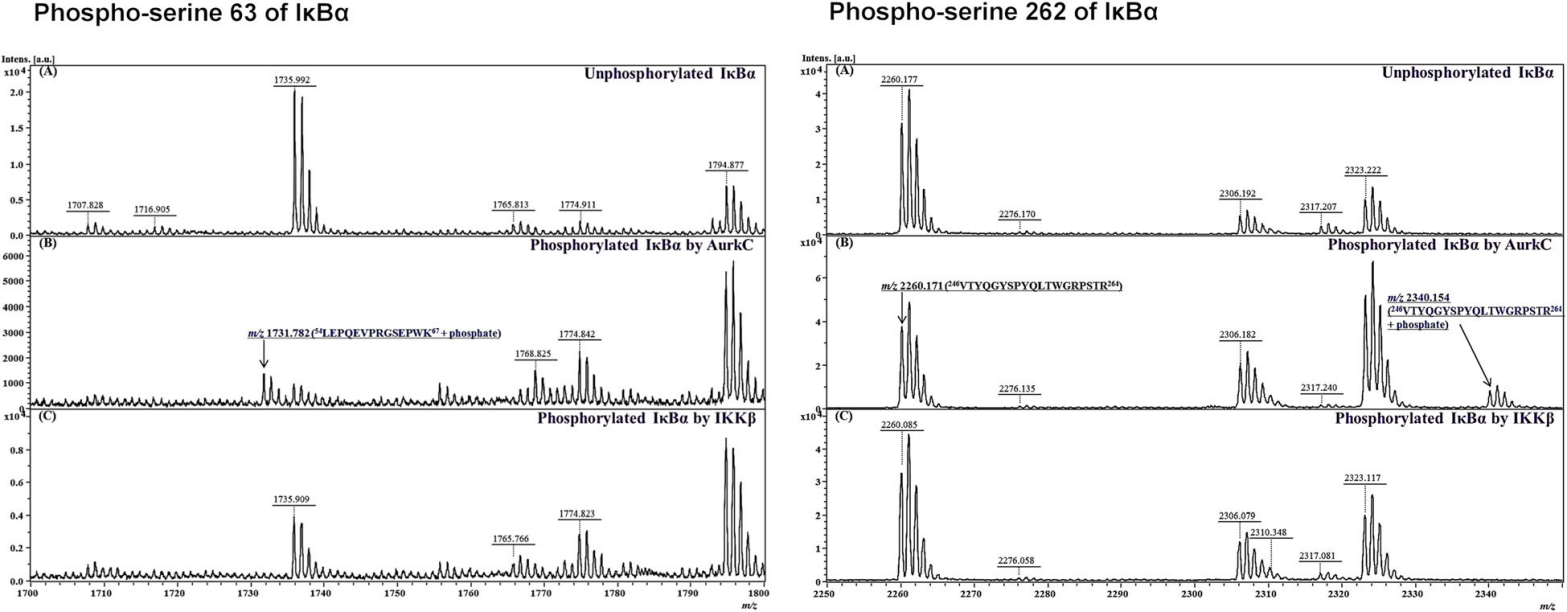
Identification of phosphorylation of serine 63 and 262 on IκBα using MALDI-TOF MS. **a** Newly found phospho-serine 63 of IκBα. Comparison of Mass range expansion of 1700–1750 *m/z* regions from the MALDI mass spectra of (**a**) inactivated IκBα; (**b**) IκBα activated by AurkC; (**c**) IκBα activated by IKKβ. Mass spectrum of IκBα activated by AurkC indicated exclusive peaks. Peaks were 54–67 peptide including phospho-serine 63 amino acid from IκBα. **b** Novel phospho-site serine 262 amino acid from IκBα**.** Comparison of MALDI MS spectra of (**a**) non-activated IκBα; (**b**) IκBα activated by AurkC; (**c**) IκBα activated by IKKβ tryptic digestion peptide between *m/z* 2250 and 2350 was shown. Unique peaks found *m/z* 2340.154 in (**b**). Peaks were 246–264 peptide including unknown phosphorylated amino acid from IκBα. A peptide present five serine and threonine

We compared the spectrum of IκBα phosphorylated by AURKC with the spectra of unphosphorylated IκBα and IκBα phosphorylated by IKKβ. We identified a novel peak that was present only in IκBα phosphorylated by AURKC (Fig. 1). The peptides at *m/z* 1731.81 (P2) and *m/z* 2340.15 (P3) were expected to increase ~ 80 Da each relative to peptides T9–10 (amino acids 54–67) and T20 (amino acids 246–264) (Supplementary Fig. 2), indicating that IκBα contains a novel AURKC phosphorylation site (Supplementary Table 2). Phosphopeptide analysis was performed in positive ion detection mode without enrichment.

### Profiling of phosphopeptides in IκBα by AurkC using nanoLC-quadrupole orbitrap MS/MS

**Fig. 2:**
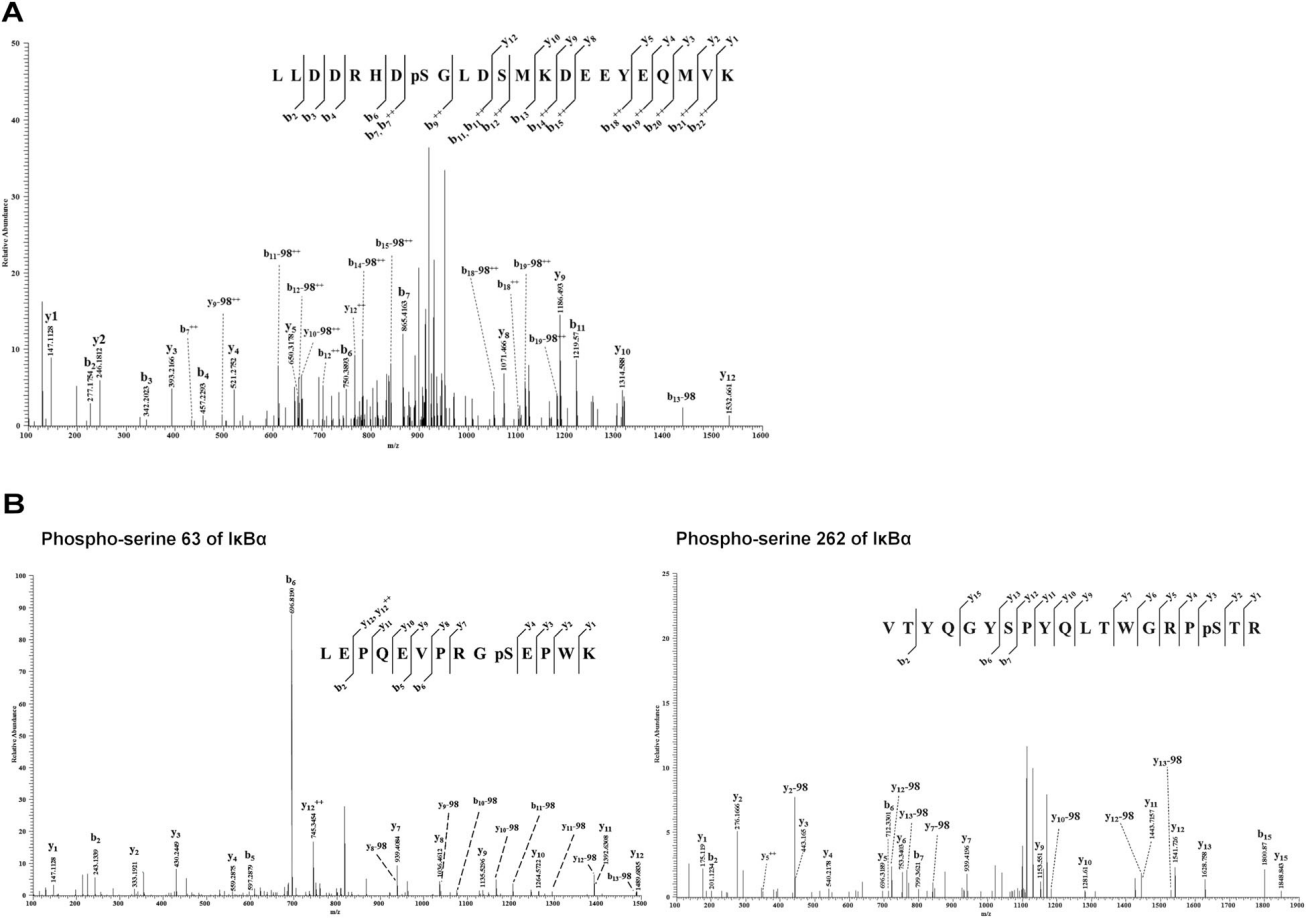
Identification of phosphorylation of serine 63 and 262 on IκBα using nanoLC-quadrupole orbitrap MS/MS. **a** Novel phosphorylated serine 63 residue of IκBα was activated by AurkC. LC-quadrupole orbitrap MS/MS spectra of precursor ion m/z 866.4101 (charge state 2^+^) representing the phospho-peptide of sequence 53-LEPQEVPRGpSEPWK-68. The presence of a y_n_, y_n_-98 and b_n_-98 series identifies phosphorylation at serine 63. **b** Novel phosphorylated serine 262 of IκBα activated by AurkC. LC-MS/MS spectra of precursor ion m/z 1170.5469 (charge state 2+) representing the phosphopeptide of sequence 245-VTYQGYSPYQLTWGRPpSTR-265

LC-quadrupole orbitrap MS/MS (LC MS/MS) analysis (Q-Exactive Plus) was performed to confirm the MALDI-TOF results. The peptide mixture was generated by an in-gel digestion process and then dissolved in 2% PA. LC MS/MS was performed to separate the tryptic peptides and identify the separated peptides. The Mascot data search program was operated under tryptic mis-cleavage 2, methionine oxidation, and serine/threonine phosphorylation conditions. The LC MS/MS data were matched to 83% of the sequence of IκBα, except that the largest molecular weight peptide (265–314; 5848.57 Da) and short LTL peptide (315–317; 346.23 Da) from the C- terminal region were produced by trypsin. Moreover, 63 phosphopeptides obtained from the Mascot search results had the expected cut-off value (10–5). Phosphorylated S32-, S63-, and S262-containing peptides of various lengths were confirmed. In conclusion, we observed S32 (Fig. 2a), S63, and S262 phosphorylation sites in IκBα using by AurkC (Fig. 2b). Phospho-S63 and phospho-S262 have not been reported in IκBα until now.

### AURK phosphorylates the new phosphorylation site of IκBα

**Fig. 3:**
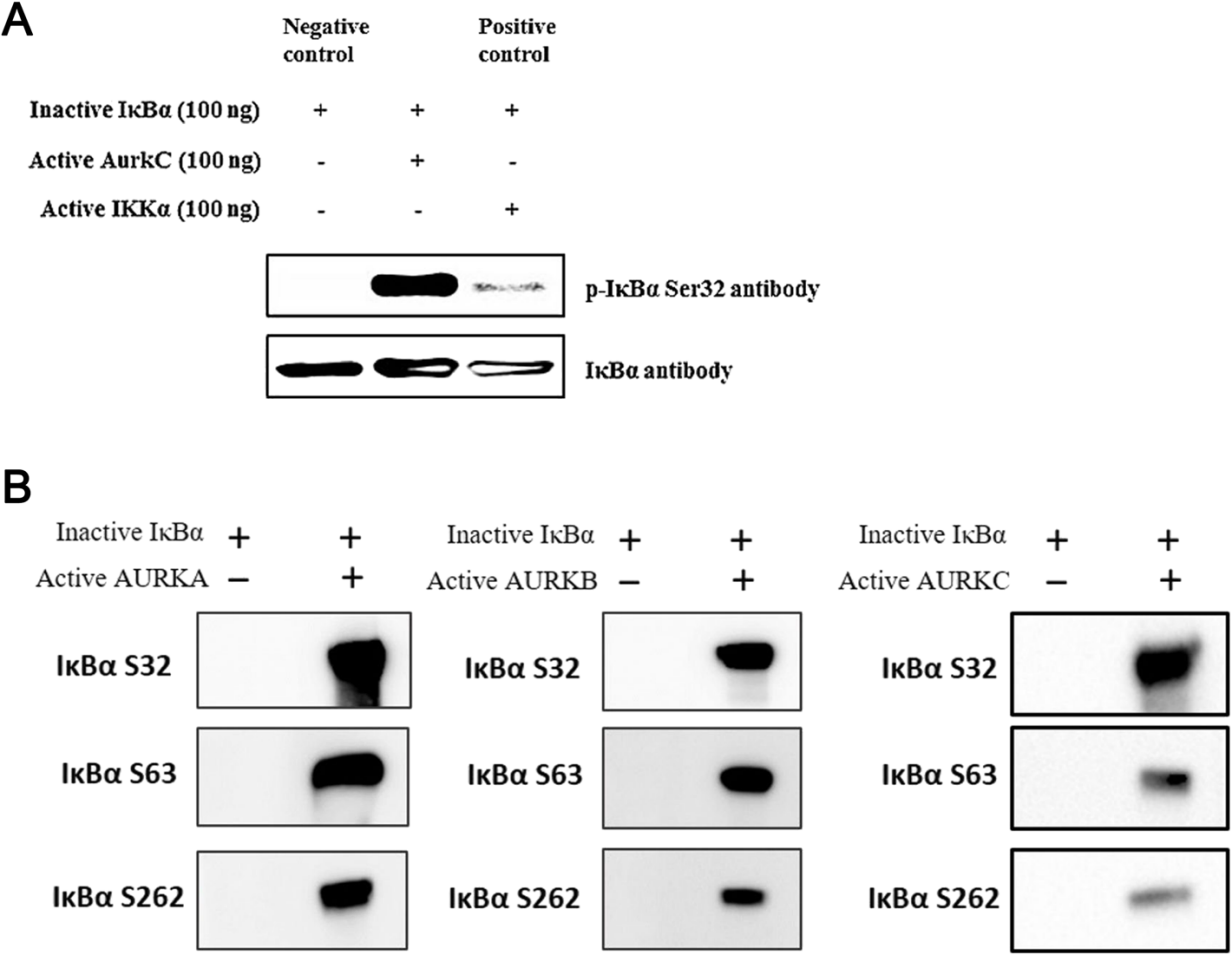
Phosphorylation of IκBα by Aurora Kinase. **a** IκBα is phosphorylated by AURKC. In vitro phosphorylation of IκBα by AURKC or IKKα kinase protein was detected using an antibody against phospho-Ser32. IκBα was phosphorylated by both AURKC and IKKα. **b** In vitro phosphorylation of IκBα by all AURK proteins was confirmed using antibodies against phospho-Ser63 and phospho-Ser262. IκBα was phosphorylated by all active AURK proteins

To confirm phosphorylation of the newly discovered S63 and S262 sites, we performed in vitro kinase assays. Because no anti–phospho-S63 or -S262 antibody was available, we raised antibodies against the corresponding phosphopeptides. First, we sought to confirm that S32 of IκBα was phosphorylated by AURK (Fig. 3a). To this end, we reacted AURKC with inactive IκBα, and found that AURKC phosphorylated IκBα at S32. Based on these results, we concluded that IκBα S63 and S262, the newly detected AURK sites, were indeed phosphorylated (Fig. 3b). All three AURK family members phosphorylated IκBα at S63 and S262, as well as at S32; affinity was highest for AURKA and lowest for AURKC. Together, these observations confirmed that the newly identified phosphorylation sites in IκBα were phosphorylated.

### Ser-63 and 262 mutations of IκBα inhibit cell proliferation in breast cancer cells

**Fig. 4:**
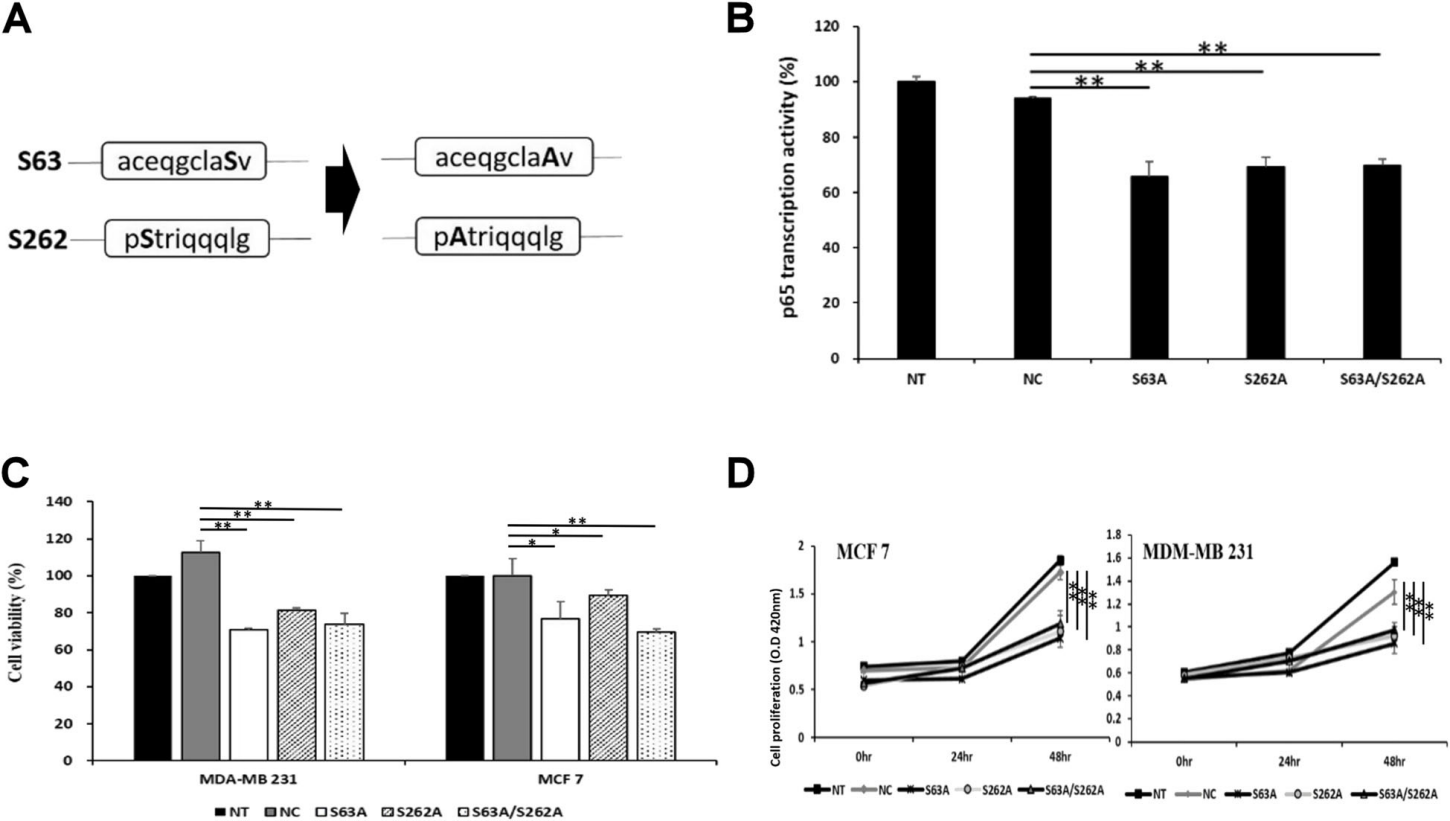
Phosphorylation of Ser-63 and 262 of IκBα plays a crucial role in cell proliferation. **a** S63A and S262A were constructed by introducing point mutations that replaced serine with alanine. **b** Transcriptional activity of p65 (i.e., activity of the NF-κB pathway) decreased in the S63A, S262A, and S63A/S262A mutants (*p < 0.05, **p < 0.001 vs. negative control). **c** Viability of the breast cancer cell lines MCF7 and MDA-MB 231 was reduced in the S63A and S262A mutants (**p < 0.001 vs. negative control). **d** Proliferation of breast cancer cell lines MCF7 and MDA-MB 231 was significantly reduced in the S63A and S262A mutants (NC vs **p < 0.001)

Novel S63 and S262 mutants were constructed by single point mutations; serine was substituted with alanine to confirm the intracellular mechanism (Fig. 4a). p65 transcriptional activity was confirmed to examine the effect of S63 and S262 phosphorylation on NF-κB activity in MDA-MB 231 cells (Fig. 4b). p65 transcriptional activity in the S63A and S262A mutants was decreased compared with that in the control, and p65 activity in the S63A mutant was further reduced compared with that in the S262A mutant. Inhibition of NF-κB activity was closely related to survival of cancer cells, confirming the viability and proliferation of the breast cancer cells. MDA-MB 231 and MCF-7 cells showed decreased tumor cell viability in the S63A and S262A mutants (Fig. 4c). Also, cell viability was decreased slightly in the S63A mutant compared to the S262A mutant. Tumor cell proliferation was also inhibited by both mutations (S63A and S262A; Fig. 4d). However, synergistic effects in the S63A and S262A double mutants were not observed in any of the three experiments. Nevertheless, the phosphorylation of IκBα at S63 and S262 not only replaced IκBα S32, but also regulated downstream NF-κB activity (Supplementary Fig. 4A). In other words, the phosphorylation of S63 and S262 functionally compensated for the absence of phosphorylation in S32.

### S63A and S262A of IκBα induced necroptosis in MDA-MB 231 cells

**Fig. 5:**
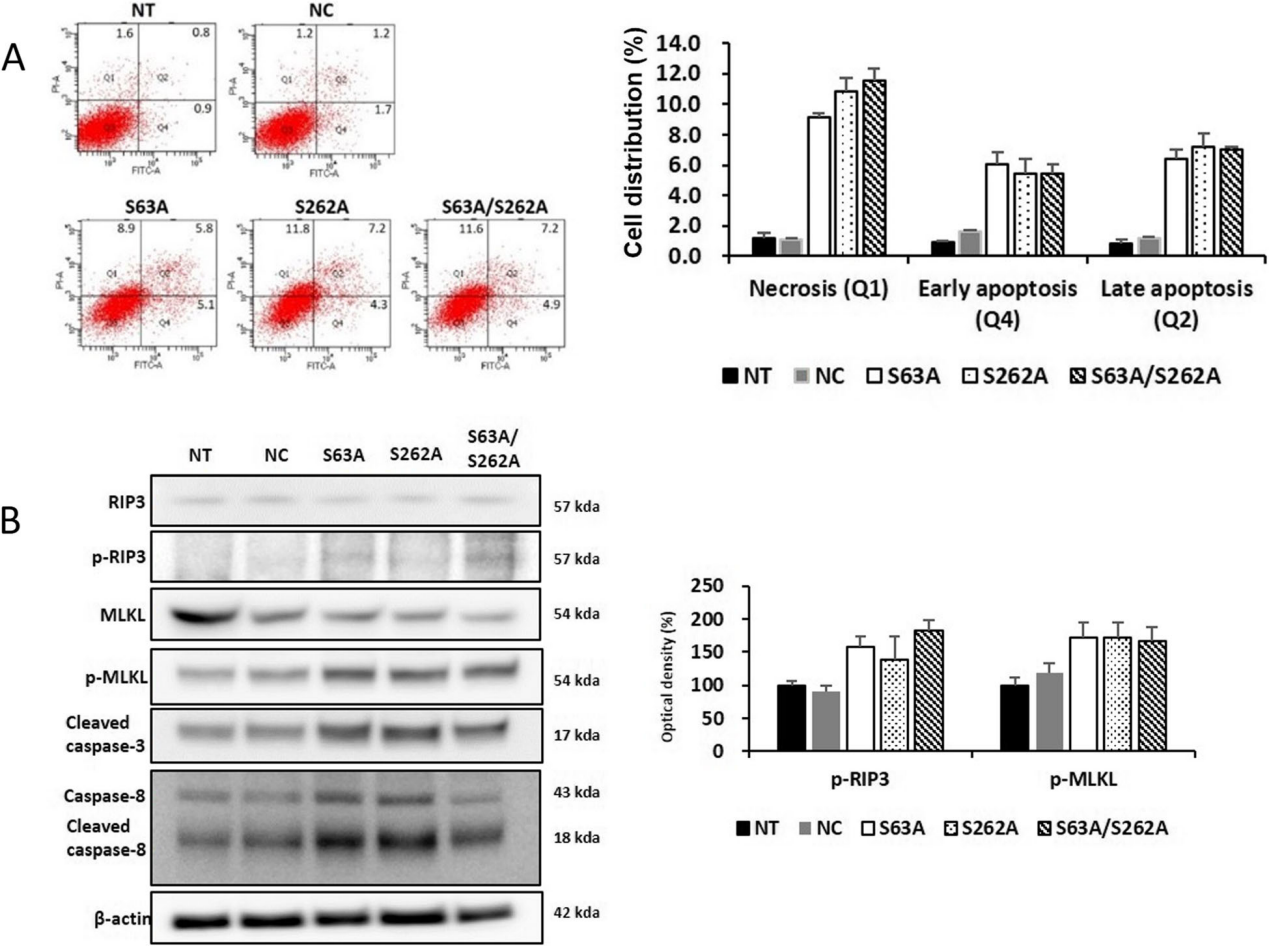
Phosphorylation of Ser-63 and 262 of IκBα plays a crucial role in necroptosis. FACs-annexin V-staining was performed to measure apoptosis in MDA-MB 231. **a** Compared with the control group, the distribution of early and late apoptosis was increased and the distribution of necrosis was increased in S63A and S262A (NC vs *p < 0.05, **p < 0.001). **b** Compared with the control group, expression of phospho-MLKL and phospho-RIP3 was increased in S63A and S262A, and expression of cleaved caspase 3 and caspase 8 was also increased

Given that single point mutations in IκBα affected the viability and proliferation of breast cancer cells, we next sought to characterize the degree of apoptosis in the mutants. We measured apoptosis in MDA-MB 231 cells harboring each mutation (Fig. 5a). Cell death increased 3–4-fold in the S63A and S262A mutants relative to the control, but we observed no significant difference between the single mutants and the double mutant. Interestingly, necrosis was more prominent than apoptosis: cells that died by apoptosis also stained positively for propidium iodide, but the cell population proceeded to Q3-Q1 as well as Q3-Q4-Q2 (Q1: Necrosis, Q2: Late Apoptosis, Q3: Early Apoptosis, Q4: Live cells). Therefore, programmed necrosis or necroptosis in the population increased for Q1. We obtained similar results in two other cell lines, MCF7 and MDA-M 468 (Supplementary Fig. 3). MDA-MB 468 exhibited more necroptosis than MCF7. RIP3 and MLKL, which are involved in necroptosis, were phosphorylated 2–2.5-fold more in the mutant cells than in the control (Fig. 5b). The mutants also contained higher levels of cleaved caspase-3 and -8 relative to the control group. Based on these results, we concluded that phosphorylation of S63 and S262 of IκBα also have important functional effects related to apoptosis and necroptosis. Moreover, TNBC cell lines harboring these mutations exhibited more necroptosis than other breast cancer cell lines.

## Discussion

NF-κB activity is mediated through the phosphorylation and degradation of IκBα, which are important intracellular mechanisms [8]. IκBα is phosphorylated by a variety of kinases [8, 18]. The most important pathway is phosphorylation of S32 and S36 by IKKβ, which leads to the degradation of IκBα [8, 18]. Other pathways include phosphorylation of IκBα by casein kinase II (CK2), which leads to the phosphorylation and degradation of IκBα at S283, S293, and Thr299 [19, 20]. However, some IκBα phosphorylation mechanisms and phosphorylation sites remain to be discovered, and many of their biological and oncological functions remain unknown.

In this study, we identified new phosphorylation sites of IκBα and obtained clues regarding its oncological function. IκBα is phosphorylated by PPIs with AURK family members [5, 17], which also regulate the cell cycle and cell division. To suppress the proliferation of cancer cells, more powerful inhibition of IκBα is required; accordingly, IκBα activity is controlled through AURK proteins [17]. The ankyrin domain of IκBα is required for the interaction with AURK. IκBα phosphorylation by AURK differs from IκBα phosphorylation by IKK family proteins; for example, the S36 residue is not phosphorylated by AURKC [17, 21]. We identified three novel phosphorylation sites at S63, S160, and S262 using MALDI-TOF MS and nanoLC-MS/MS [22, 23]. However, in vitro phosphorylation assays revealed modification at Ser63 and Ser262. Ser160 was not only weakly phosphorylated, as revealed by an in vitro kinase assay, but also exhibited reduced antibody specificity (data not shown). The in vitro kinase assay revealed that Ser63 and Ser262 of IκBα were phosphorylated as much as Ser32 by AURK. Minor phosphorylation is known to be inferior to most major phosphorylation mechanisms, such as biological or oncological functions. The sites we identified in this study (S63 and S262) were affected to a lesser degree than S32 (Supplementary Fig. 4), but the phosphorylation event was catalyzed by AURK, not the previously known NF-kappaB pathway. We also confirmed that the proliferation and viability of breast cancer cells were decreased in cell lines carrying either of two amino acid substitutions, S63A and S262A, suggesting that alanine substitution suppressed the degradation of IκBα (Supplementary Fig. 4) and inhibited breast cancer cell proliferation and survival. Although p65 activity was inhibited in mutants, the double mutant had no additive or synergistic effect. In structural terms, both S63 and S262 of IκBα are located near the interface between IκBα and NF-κB p65, so it is unlikely that the double mutant would have a synergistic effect (https://www.rcsb.org/3d-view/1IKN). To confirm the reduction in breast cancer cell survival, we performed a FACS-based apoptosis assay. Both the S63A and S262A mutants exhibited an increase in early and late apoptosis. Interestingly, necrosis was more prominent than apoptosis. Based on these results, we monitored the expression and phosphorylation of RIP3 and MLKL, which are involved in necroptosis and programmed necrosis, in the S63A and S262A mutants. The phosphorylation of both proteins was elevated in both mutants. IκBα, a major cellular regulator, is regulated through phosphorylation by various kinases [11, 12]. In particular, avoiding necroptosis or apoptosis through phosphorylation by AURK is an important regulatory mechanism in cancer cells [24, 25]. The newly identified IκBα phosphorylation sites are important players in the regulation of IκBα, including necroptosis or apoptosis. Although several tumor regulatory mechanisms remain to be identified, the discovery of this phosphorylation mechanism provides important insights into the regulation of breast cancer.

## Conclusions

We identified novel phosphorylation sites of IκBα by AURK, and its sites were related to apoptosis. But, the role of apoptosis in the new phosphorylation sites was insufficient compared to S32 of IκBα. However, based on the results of FACs, it was newly identified as a site involved in necroptosis.

## Supplementary Information

**Additional file 1: Supplementary Figure 1.** Schematic diagram of phosphopeptide mapping. Schematic diagram was constructed for phosphopeptide mapping. **Supplementary Figure 2.** MALDI MS spectrum of IκBα tryptic digestion products after phosphorylation with AURKC. Peaks corresponding to phosphopeptides and peptides are marked with peptide IDs of Table 2 and fragments of Table 1. **Supplementary Figure 3.** Ion chromatograms of digested phosphopeptide including Serine 32. LC-quadrupole orbitrap MS/MS spectrum of precursor ion m/z 950.4053 (charge state 3+) representing the phosphorylated peptide of sequence 24-LLDDRHDpSGLDSMKDEEYEQMVK-48. The spectrum exhibits evidence of phosphorylation at serine 32, but not serine 36. **Supplementary Figure 4.** Inhibition of cell proliferation and apoptosis in MDM-MB231 expressing the S32A mutant. p65 transcriptional activity in MDA-MB 231 was measured by ELISA. A) Relative to NC, the S32A mutant had the strongest inhibitory effect on transcriptional activity. Similar suppression was confirmed for S63A and S262A (*p < 0.05, **p < 0.001 vs. negative control). B, C) S32A, the positive control, had a stronger effect on cancer cell proliferation and cell death than the other mutants (*p < 0.05, **p < 0.001 vs. negative control). **Supplementary Table 1.** Identification of a novel phosphorylation site by LC-MS. New phosphorylation sites were identified between amino acids 54–67 and 246–264. **Supplementary Table 2.** Confirmation of novel phosphorylation site by MALDI-TOF MS. New phosphorylation sites were confirmed using MALDI-TOF MS.

## Data Availability

The datasets used and/or analyzed during the current study are available from the corresponding author on reasonable request. All data generated or analyzed during this study are included in this published article (and its supplementary information files).

## Abbreviations

MS: Mass spectrometry
MALDI: Matrix-assisted laser desorption/ionization
TOF: Time of flight
LC: Liquid Chromatography
IP: Immunoprecipitation
IκBα: nuclear factor of kappa light polypeptide gene enhancer in B cells inhibitor, alpha
AURK: Aurora Kinase
IKK: IκB kinase
TEV protease: Tobacco etch virus protease
CAN: Acetonitrile
SDS-PAGE: Sodium Dodecylsulfate Polyacrylamide Gel electrophoresis
2,5-DHB: 2,5-dihydroxybenzoic acid
PA: phosphoric acid
TUNEL: terminal deoxynucleotidyl transferase dUTP nick end labeling
PPI: Protein-Protein Interaction
