# Correction to: Co-relation with novel phosphorylation sites of IκBα and necroptosis in breast cancer cells

**DOI:** 10.1186/s12885-021-08919-w

**Published:** 2021-11-11

**Authors:** Sung Hoon Choi, Hee-Sub Yoon, Shin-Ae Yoo, Sung Ho Yun, Joo-Hee Park, Eun Hee Han, Sung-Gil Chi, Young-Ho Chung

**Affiliations:** 1 Research Center for Bioconvergence Analysis, Korea Basic Science Institute (KBSI), 162 Yeongudanji-ro, Ochang-eup, Cheongwon-gu, 28119 Cheongju-si, Republic of Korea; 2 Yonsei Liver Center, Yonsei University College of Medicine, Seoul, 03722 South Korea; 3 Department of Life Sciences, Korea University, Seoul, 02841 Republic of Korea; 4 GRAST, Chungnam National University, Daejeon, 34134 Republic of Korea

**Correction to: BMC Cancer 21, 596 (2021)**

**https://doi.org/10.1186/s12885-021-08304-7**

Following publication of the original article [1], the authors identified an error in the contribution section of this article. Sung Hoon Choi and Hee-Sub Yoon contributed equally to this work and are considered co-first authors.

The author contribution has been updated above and the original article [1] has been corrected.

## References

[1] Choi S, Yoon HS, Yoo SA (2021). Co-relation with novel phosphorylation sites of IκBα and necroptosis in breast cancer cells. BMC Cancer.

