# Supplementary material for: Co-relation with novel phosphorylation sites of IκBα and necroptosis in breast cancer cells
Source: BMC Cancer. 2021 May 24;21:596. doi: 10.1186/s12885-021-08304-7 (PMC8147041; doi:10.1186/s12885-021-08304-7)
Supplement: Supplementary file 1 — Additional file 1: Supplementary Figure 1. Schematic diagram of phosphopeptide mapping. Schematic diagram was constructed for phosphopeptide mapping. Supplementary Figure 2. MALDI MS spectrum of IκBα tryptic digestion products after phosphorylation with AURKC. Peaks corresponding to phosphopeptides and peptides are marked with peptide IDs of Table 2 and fragments of Table 1. Supplementary Figure 3. Ion chromatograms of digested phosphopeptide including Serine 32. LC-quadrupole orbitrap MS/MS spectrum of precursor ion m/z 950.4053 (charge state 3+) representing the phosphorylated peptide of sequence 24-LLDDRHDpSGLDSMKDEEYEQMVK-48. The spectrum exhibits evidence of phosphorylation at serine 32, but not serine 36. Supplementary Figure 4. Inhibition of cell proliferation and apoptosis in MDM-MB231 expressing the S32A mutant. p65 transcriptional activity in MDA-MB 231 was measured by ELISA. A) Relative to NC, the S32A mutant had the strongest inhibitory effect on transcriptional activity. Similar suppression was confirmed for S63A and S262A (*p < 0.05, **p < 0.001 vs. negative control). B, C) S32A, the positive control, had a stronger effect on cancer cell proliferation and cell death than the other mutants (*p < 0.05, **p < 0.001 vs. negative control). Supplementary Table 1. Identification of a novel phosphorylation site by LC-MS. New phosphorylation sites were identified between amino acids 54–67 and 246–264. Supplementary Table 2. Confirmation of novel phosphorylation site by MALDI-TOF MS. New phosphorylation sites were confirmed using MALDI-TOF MS. [file 12885_2021_8304_MOESM1_ESM.pdf]

Supplementary Figure 1

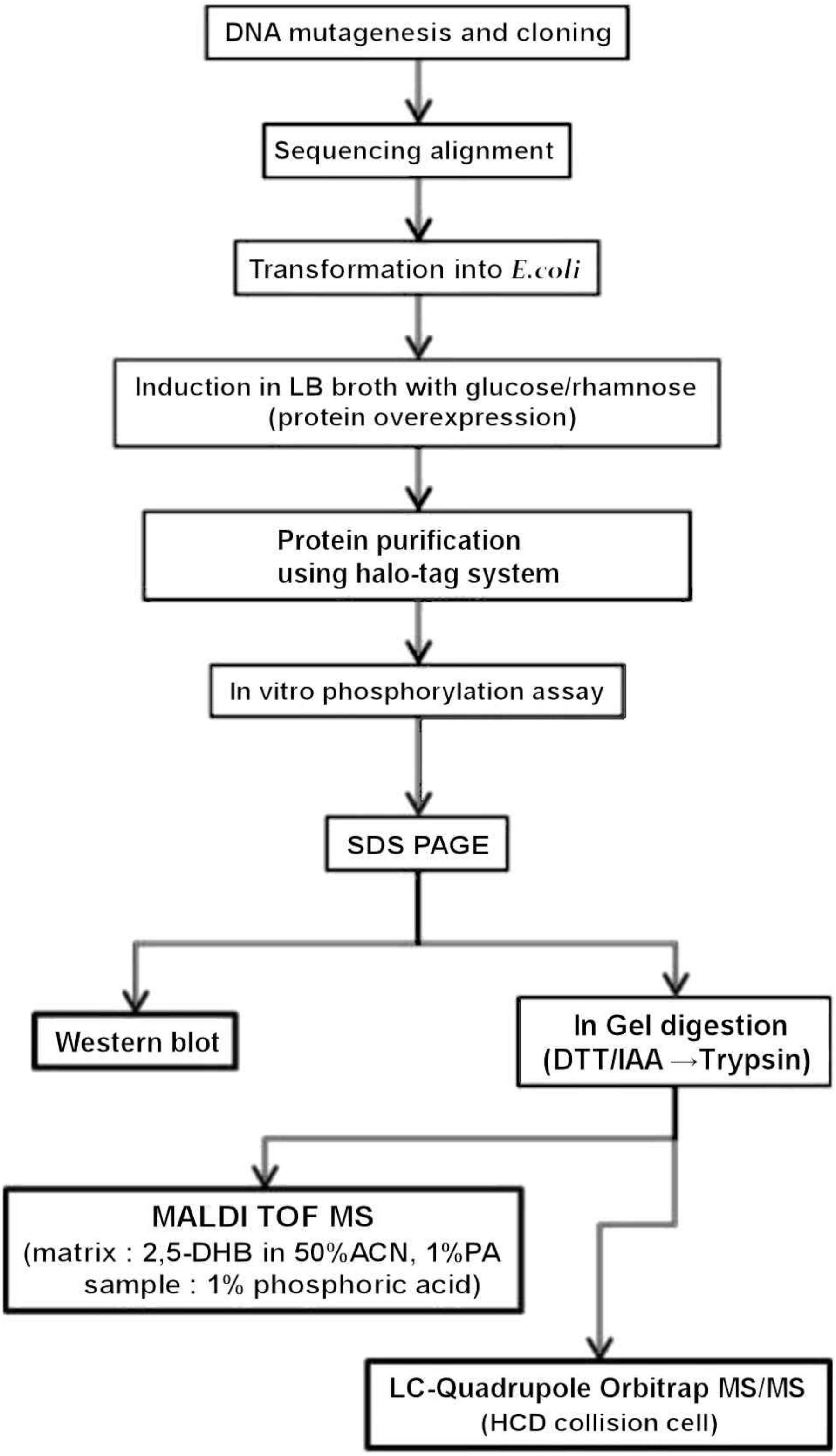

Supplementary Figure 2

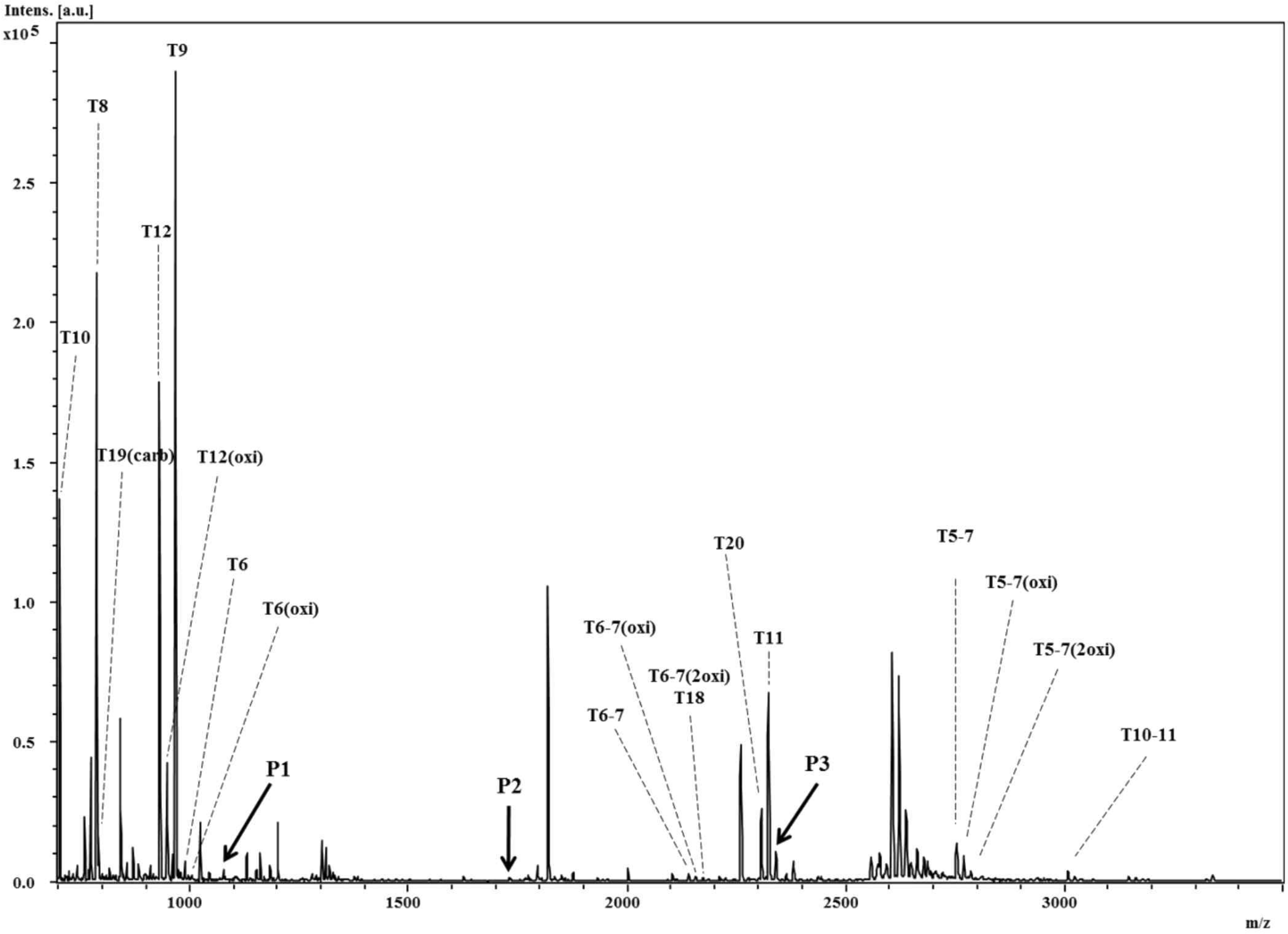

Supplementary Figure 3

MCF 7

NT NC S63A S262A

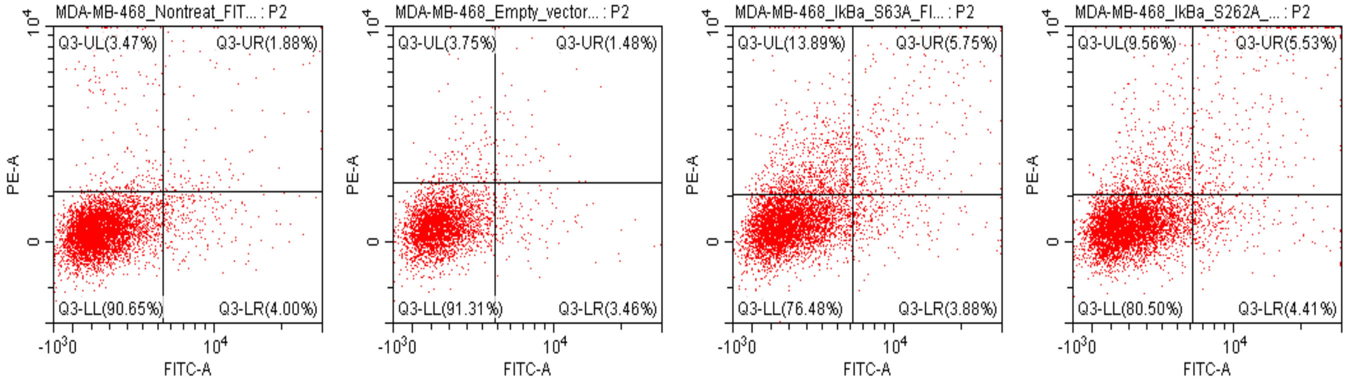

MDA-MB 468

NT NC S63A S262A

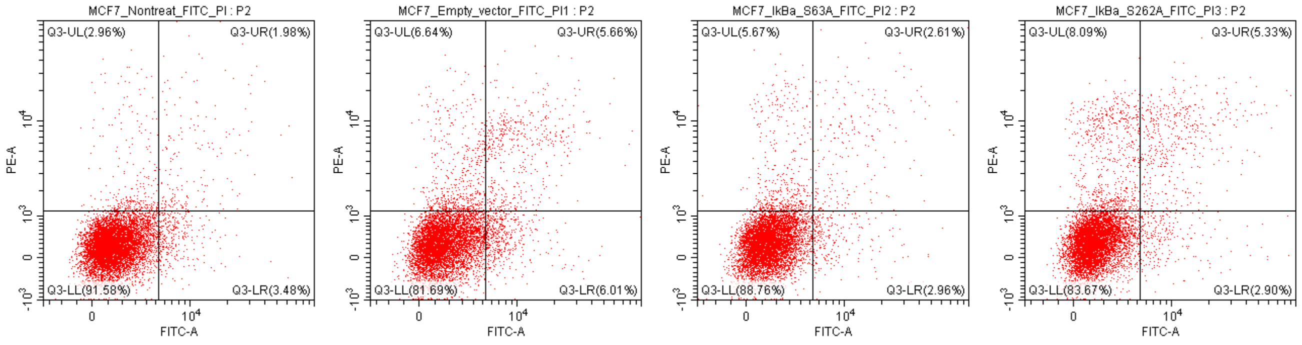

Supplementary Figure 4

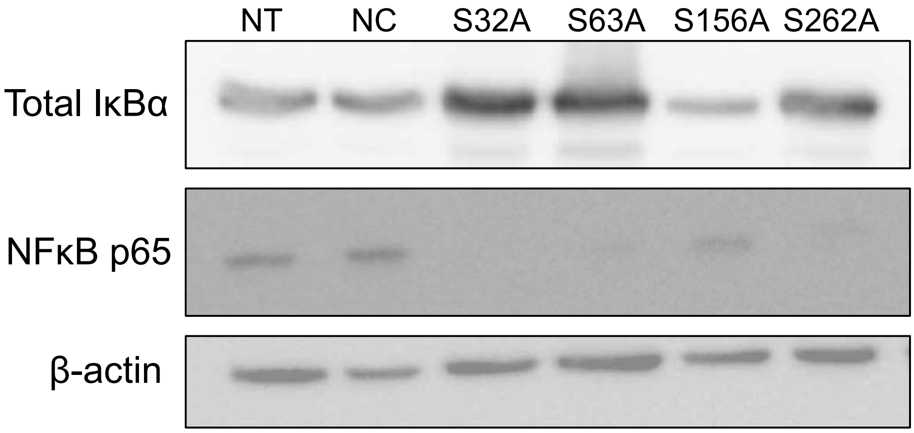

Supplementary Table 1

Peptide ID	Sequence	AA No.	MC	Phospho -site	confirm	Mass MH^+	
						Theoretical mass	Experimental m/z value
P1	HDpSGLDSMK	30-38	0	S32	western blot	1069.4019	1069.482
P2	LEPQEVPR GpSEPWK	54-67	1	S63	LC-MS/MS	1731.8101	1731.782
P3	VTYQGYSPYQLTWGRPpSTR	246-264	0	S262	LC-MS/MS	2340.0808	2340.154

Supplementary Table 2

AA no.	MC	Phospho-site	MH^+	Charge states	Ions Score	Sequence
25-47	2	S32	2832.1983	3+	66	LLDDR HDpSGLDSMK DEEYEQMVK
48-67	2	S63	2499.2159	3+	52	ELQEIR LEPQEVPR GpSEPWK
54-67	1	S63	1730.8029	2+	67	LEPQEVPR GpSEPWK
54-87	2	S63	4034.9415	3+	57	LEPQEVPR GpSEPWK QQLTEDGDSFLHLAIIHEEK
62-87	1	S63	3086.4386	3+, 4+	75	GpSEPWK QQLTEDGDSFLHLAIIHEEK
144-177	0	S160	3721.778	4+	75	GNTPLHLACEQGCLApSVGVLTQSCTTPHLHSILK
239-264	1	S262	3111.4022	3+	57	CGADVNR VTYQGYSPYQLTWGRPpSTR
246-264	0	S262	2340.0808	2+, 3+	91	VTYQGYSPYQLTWGRPpSTR
